# Acacetin Protects Myocardial Cells against Hypoxia-Reoxygenation Injury through Activation of Autophagy

**DOI:** 10.1155/2021/9979843

**Published:** 2021-06-29

**Authors:** Chong Liu, Minmin Zhang, Shenyi Ye, Chenliang Hong, Jiaxi Chen, Ruyue Lu, Bingjie Hu, Weijun Yang, Bo Shen, Zhengyi Gu

**Affiliations:** ^1^Taizhou Hospital of Zhejiang Province Affiliated to Wenzhou Medical University, 317000 Taizhou, China; ^2^Enze Hospital, Taizhou Enze Medical Center (Group), 317000 Taizhou, China; ^3^YiLi Normal University, College of Chinese Language and Literature, YiLi, 835000 Xinjiang, China; ^4^Xinjiang Institute of Materia Medica, Uygur, Xinjiang Province 830004, China

## Abstract

Ischemic heart disease is a leading cause of mortality and morbidity worldwide. We previously demonstrated that acacetin protects against myocardial ischemia reperfusion injury in rats, although the underlying mechanism remains to be elucidated. In the present study, we investigated the effects of acacetin on autophagy during hypoxia/reoxygenation (H/R) injury by exposing H9c2 myocardial cells to H/R with or without acacetin pretreatment during hypoxia. Our results show that acacetin significantly increased cell viability in a dose-dependent manner, enhanced antioxidant capacity, and suppressed protein apoptosis of rat cardiomyocytes H9c2 cells following H/R injury. In addition, lentiviral infection of H9c2 cardiomyocytes revealed that acacetin pretreatment significantly enhanced the fluorescence intensity of autophagy proteins Beclin 1, LC3-II, and p62. These results indicate that acacetin protected H9c2 cardiomyocytes from H/R damage by enhancing autophagy. Moreover, we found that application of acacetin increased activation of the PI3K/Akt signaling pathway, whereas cotreatment with the PI3K inhibitor LY294002 reversed the inhibition of apoptosis and autophagy induced by acacetin. In conclusion, acacetin mitigated H/R injury by promoting autophagy through activating the PI3K/Akt/mTOR signaling pathway.

## 1. Introduction

Ischemic heart disease is a leading cause of mortality and morbidity worldwide [1]. Following an ischemic attack, reperfusion therapy is regarded as the most effective measure to save ischemic myocardium [2]. However, myocardial ischemic-reperfusion therapy may induce ultrastructural damage and functional impairment of cardiomyocytes, which aggravates ischemic myocardium injury [3–5]. Although reports describing therapeutic interventions and prognosis of myocardial injury have recently increased, available therapies still induce ultrastructural damage and functional impairments in cardiomyocytes [6]. Many researchers have discussed problems with optimizing myocardial injury therapy, but potential mechanisms and drug targets of myocardial injury remain to be elucidated. Thus, novel pharmacological or other effective targets are urgently needed to protect against myocardial hypoxia-reoxygenation (H/R) injury.

Acacetin, an effective constituent of the Chinese traditional herb *Ziziphora bungeana* (Juz.), was previously demonstrated to protect against myocardial ischemia reperfusion injury [7, 8]. However, the effective targets and mechanism of acacetin remain to be elucidated. Autophagy is a highly conserved catabolic process involving the degradation and recycling of macromolecules, which is important for both organelle formation and lysosome degradation [9]. Moreover, autophagy has a major role in the pathological process of myocardial infarction. As cardiac tissue is comprised of terminally differentiated cardiomyocytes, autophagy occurs at a basal level under normal conditions to maintain intracellular homeostasis by removing long-lived or excess protein aggregates and damaged organelles [9–12]. Hence, we hypothesized that acacetin may protect myocardial cells against H/R injury through autophagy and investigated its targets and potential mechanisms.

Recent studies show that the phosphoinositide 3 kinase (PI3K)/Akt/mechanistic target of rapamycin (mTOR) pathway plays crucial roles in regulating mitophagy and mitochondrial quality control [13–15]. Mitophagy may have restorative potential for myocardial H/R injury [13]. However, the ability of acacetin to induce autophagy effects and related signaling pathways is poorly understood. Thus, the purpose of this study was to determine whether the molecular mechanisms underlying the cardioprotective effects of acacetin are related to promotion of autophagy via activation of PI3K/Akt/mTOR signaling.

## 2. Materials and Methods

### 2.1. Materials

Acacetin (purity≧98%) was purchased from the Xinjiang Urumqi Uygur Medicine Institute (Xinjiang, China).

### 2.2. Cell Culture

H9c2 cells exhibiting good growth were inoculated into 25 cm^2^ flasks in low-glucose Dulbecco's Modified Eagle's Medium containing 10% fetal bovine serum and placed in an incubator at 37°C with 5% CO_2_. When cell cultures reached 85%–90% confluence, they were washed twice with precooled phosphate-buffered saline (PBS), and then, the cells were digested from the culture flask with a 1 : 2 volume of 0.25% trypsin. Cells were passaged once every 2 to 3 days. Cells were inoculated into the corresponding culture plates during logarithmic growth phase [16].

### 2.3. Detection of Acacetin Cytotoxicity

Cells in logarithmic growth phase were prepared into cell suspensions that were subsequently inoculated into 96-well plates. To seed approximately 1–2 × 10^4^ cells per well, approximately 100 *μ*l of cell suspension was added into wells; one sample generated 4–6 replicates. Plates were then placed in a 37°C incubator to allow the cells to stably adhere. Subsequently, cells were incubated a gradient of acacetin concentrations (1.56, 3.13, 6.25, 12.50, 25.00, 50.00, 100.00, and 200.00 *μ*g/ml) for 12, 24, and 36 h to observe cell growth. Cell Counting Kit 8 (CCK-8; Wuhan Boster Biotechnology, Wuhan, China) reagents were nontoxic to cells and reduced by the electron carrier to orange-deficient formazan by some dehydrogenases in the mitochondria of cells. Cell viability assay is according to a previously described protocol [16].

### 2.4. Establishment of a Stimulated H/R Model in H9c2 Cardiomyocytes

To mimic H/R-induced injury *in vitro*, ischemia followed by reperfusion was stimulated in H9c2 cells [17]. After transferring cells to a closed container with an oxygen-deficient zone (Mitsubishi Gas Chemical, Tokyo, Japan) for incubation in a 5% CO_2_ incubator at 37°C to induce anoxia, cells were exposed to 4 h of simulated ischemia. Subsequently, the culture medium was replaced with normal medium, and cells were incubated in a 5% CO_2_ incubator at 37°C for 4 h to simulate reperfusion [18].

### 2.5. Detection of SOD, LDH, and MDA

Cell samples were homogenized to extract enzymes for detection. Superoxide dismutase (SOD; Nanjing Institute of Bioengineering, Nanjing, China) and malondialdehyde (MDA; Nanjing Institute of Bioengineering), and lactate dehydrogenase (LDH, Nanjing Institute of Bioengineering) activities were detected by kits purchased from the Nanjing Institute of Bioengineering according to the manufacturer's directions.

### 2.6. Western Blotting

After 12 h treatment with varying concentrations of acacetin, simulation of ischemia for the final 4 h, cells were washed twice with cold PBS and immediately lysed in radioimmunoprecipitation assay lysis buffer (Wuhan Boster Biotechnology) supplemented with phenylmethylsulfonyl fluoride and protease and phosphatase inhibitor cocktail (Cat. No. P1260; Solarbio, Beijing, China). The resulting protein was boiled for 5 min in 1× loading buffer and electrophoresed according to a previously described method [19]. Following electrophoretic separation, protein samples were transferred to polyvinylidene fluoride membranes (Millipore, Burlington, MA, USA) and blocked with 3% bovine serum albumin for 2 h [20]. Subsequently, membranes were incubated with the following primary antibodies overnight at 4°C [14]: Bax [1 : 1000; Cat. No. 2772; Cell Signaling Technology (CST), Danvers, MA, USA], p-AKT (Ser473) (1 : 2000; Cat. No. 4060, CST), Bcl-2 (1 : 1000; Cat. No. abs131701; Absin, Shanghai, China), mTOR (1 : 1000; Cat. No. 2983, CST), PI3K (1 : 1000; Cat. No. 4257, CST), Beclin 1 (1 : 1000; Cat. No. abs117840, Absin), and LC3B (1 : 1000; Thermo Fisher, Waltham, MA, USA). After washing three times, membranes were incubated with goat anti-rabbit IgG-FITC (1 : 100; abs20004ss, Absin) and goat anti-rabbit IgG-HRP (1 : 5000; abs2004ss, Absin) for 2 h at room temperature (RT). Next, membranes were washed three times with Tris-buffered saline containing Tween [21] and subsequently developed using an enhanced chemiluminescence reagent. Protein expression levels were normalized to the level of *β*-actin in corresponding lanes using Image Lab™ Version 3.0 (Bio-Rad, Hercules, CA, USA) and ImageJ 1.41 (https://imagej.nih.gov/ij/) for densitometric analysis.

### 2.7. Flow Cytometric Analysis of Apoptosis

Annexin V, FITC, and PI are sensitive probes for identifying apoptotic cells. According to our previously described method [22], each group of cells was prepared for flow cytometry. Briefly, H9c2 cells were harvested by dissociation with trypsin-EDTA, washed twice with cold PBS, and then resuspended in 1× binding buffer [23]. Next, 100 *μ*l of the solution (1 × 10^5^ cells) were transferred into a 5 ml culture tube. After adding 5 *μ*l of Annexin V-FITC and 10 *μ*l of PI, cells were gently vortexed and incubated for 15 min at RT (25°C) in the dark. Finally, 400 *μ*l of 1× binding buffer was added to each tube, and cells were analyzed by flow cytometry within 1 h [24].

### 2.8. Visualization of Autophagic Fluorescence Intensity by Microscopy

Autophagic flux in primary mouse cardiomyocytes was monitored using stubRFP-sensGFP-LC3 lentivirus, a LC3 double-fluorescent lentivirus autophagy detection system optimized for monitoring of cell autophagy by flow cytometry. The fusion protein consists of a red fluorescent protein (Stub-RFP), green fluorescent protein (Sens-GFP), and autophagy-labeled protein LC3 [25]. Cardiomyocytes were transfected with stubRFP-sensGFP-LC3 lentivirus and seeded onto six-well plates. Twelve hours later, cardiomyocytes were exposed to different experimental conditions (control, H/R, or H/R + Acacetin). Cells were then analyzed with a U-LH100HG optical microscope (Olympus, Tokyo, Japan) [20].

### 2.9. Statistical Analyses

Statistical analysis was conducted with SPSS 22.0 software (IBM, Armonk, NY, USA. Data are expressed as mean ± standard deviation. *P* < 0.05 was considered to be statistically significant.

## 3. Results

### 3.1. Cell Viability

**Figure 1:**
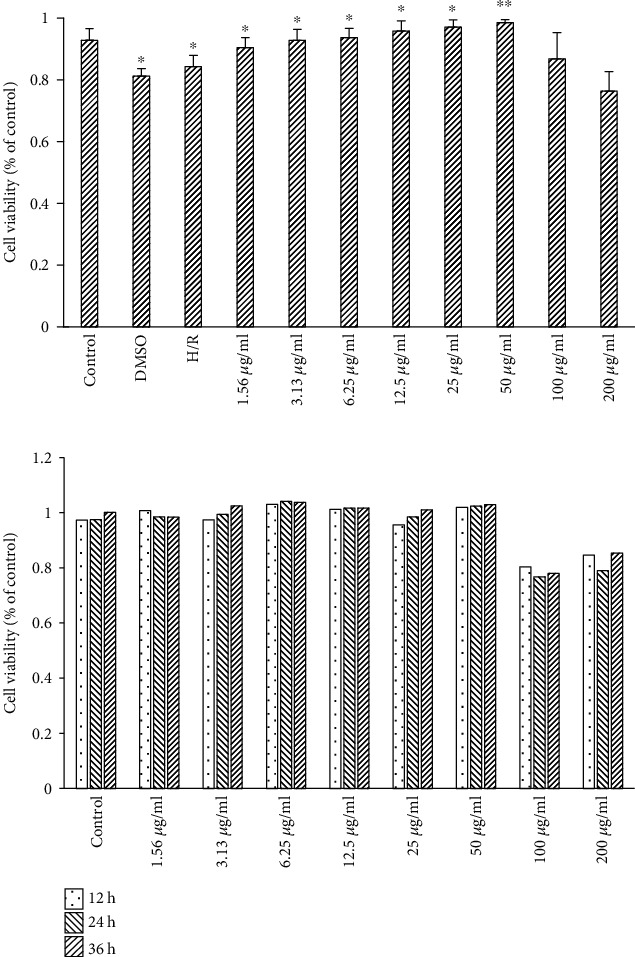
Effect of acacetin on viability of H9c2 cells. (a) Effect of acacetin on viability of H9c2 cells was detected by CCK-8 assay. (b) Effect of varying pretreatment time (12, 24, and 36 h) with acacetin on viability of H9c2 cells was detected by CCK-8 assay. Bars indicate mean ± standard deviation of three independent experiments. CCK-8: Cell Counting Kit 8.

We first assayed the effect of acacetin on cell viability and found that viability gradually increased with increasing concentrations of acacetin in the range of 1.56–50 *μ*g/ml. These results indicate that the activity elicited by acacetin occurred in a concentration-dependent manner. When the concentration of acacetin reached 100–200 *μ*g/ml, cell viability was slightly decreased (Figure 1(a)). In addition, we further evaluated the effect of varying acacetin pretreatment time on the viability of cardiomyocytes. The rate of cell survival was the highest at 12 h, but decreased at 24 h and 36 h; thus, 12 h was selected as the pretreatment time for follow-up experiments (Figure 1(b)).

### 3.2. Acacetin Enhanced the Antioxidant Capacity of Cardiomyocytes

**Figure 2:**
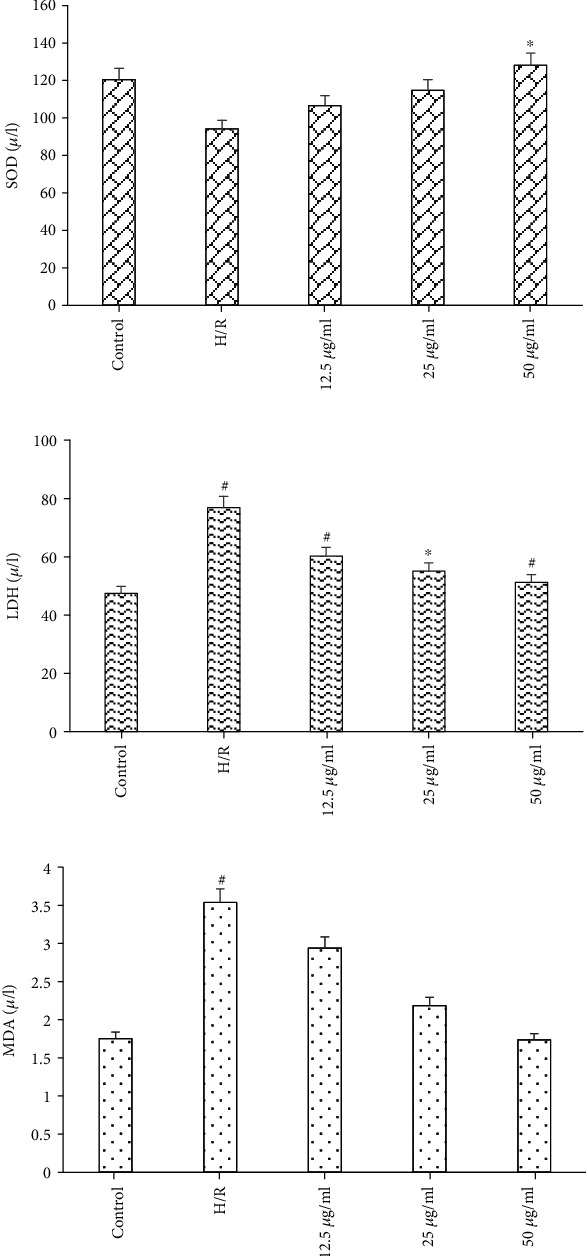
Release of SOD, LDH, and MDA at the end of H/R was determined. Results for control, H/R model, and H/R + acacetin (12.5, 25, and 50 *μ*g/ml) groups are expressed as percentages of control and presented as mean ± standard deviation of five independent experiments. ^#^*P* < 0.05 compared with control group; ^#^*P* < 0.05 vs. ^∗^*P* < 0.01 compared with H/R group. H/R: hypoxia/reoxygenation; LDH: lactate dehydrogenase; MDA: malondialdehyde; SOD: superoxide dismutase.

Antioxidant enzymes are important indicator of cardiac antioxidant capacity. Therefore, we investigated the effect of acacetin on protein levels of reactive antioxidant enzymes in cardiomyocytes. Protein levels of SOD, an important antioxidant enzyme [26], were significantly decreased by H/R but increased with acacetin pretreatment (Figure 2(a)). In addition, the activities of MDA and LDH were reduced in acacetin pretreatment groups, indicating that cell injury was inhibited by acacetin pretreatment (Figures 2(b) and 2(c)). This effect was most significant at concentration of 50 *μ*g/ml acacetin, consistent with previous experimental results. These results indicate that following H/R, a large number of oxygen free radicals were released, biofilm lipidization was severe, and cell membrane permeability was increased [27], resulting in significant increases of LDH and MDA activities and a significant decrease of SOD activities compared with the control group.

### 3.3. Acacetin Inhibited H/R-Induced Cell Apoptosis

**Figure 3:**
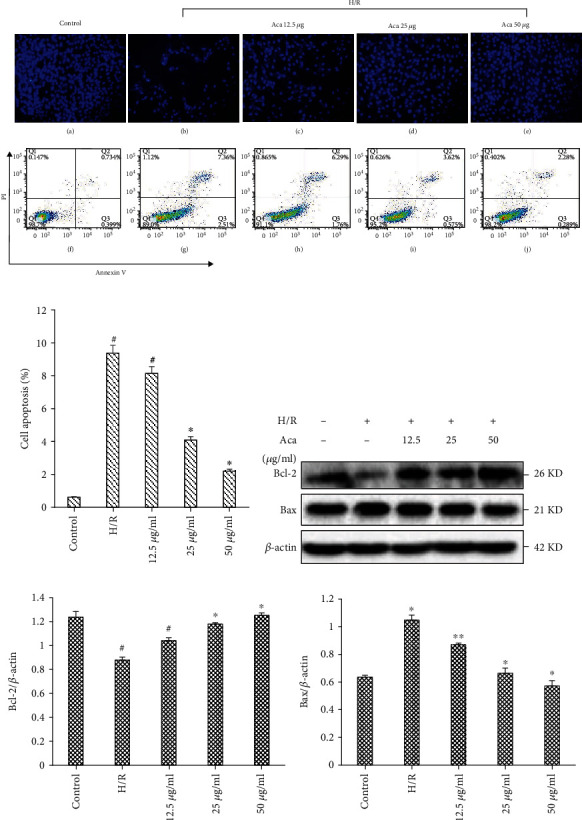
Acacetin suppresses apoptosis of H9c2 cells in H/R injury. H9c2 cells were divided into control, H/R model, and H/R + acacetin (12.5, 25, and 50 *μ*g/ml) groups. (a) Cell apoptosis rates of each group were analyzed by flow cytometry, while cell survival rates were analyzed by DAPI staining. (b–d) Relative protein levels of cleaved Bax and Bcl-2 in each group were measured by Western blot. Bars indicate mean ± standard deviation of three independent experiments. ^#^*P* < 0.05 compared with control group; ^#^*P* < 0.05, ^∗^*P* < 0.01 compared with H/R model group. DAPI: 4′,6-diamidino-2-phenylindole; H/R: hypoxia/reoxygenation.

We further examined the effects of acacetin on cardiomyocytes following H/R injury by flow cytometry and 4′,6-diamidino-2-phenylindole (DAPI) staining. Both results showed that acacetin could inhibit cell apoptosis induced by H/R injury (Figures 3(a) and 3(b)). In addition, we evaluated Bcl-2 and Bax expression after H/R injury, and the protection elicited by acacetin. Our results show that compared with the H/R model group, Bax protein expression was significantly decreased, and Bcl-2 protein expression was significantly increased in cells pretreated with acacetin for 12 h (Figures 3(c)–3(e)). These results suggest that the inhibitory effect of acacetin on cardiomyocyte apoptosis may be achieved by increasing Bcl-2 expression and inhibiting Bax activation.

### 3.4. Acacetin Induced Autophagy to Protect Cardiomyocytes against H/R Injury

**Figure 4:**
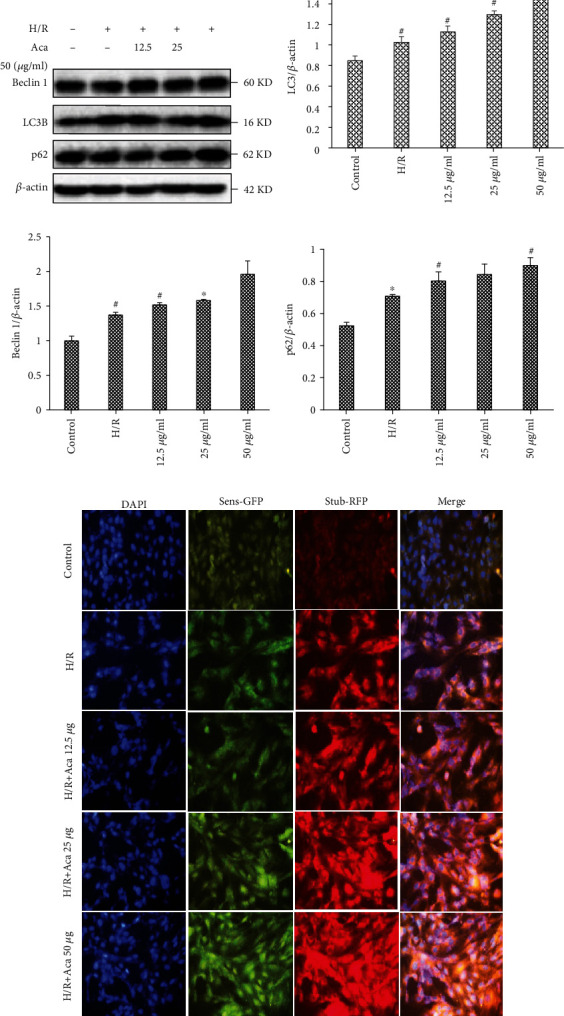
Acacetin promotes autophagy to suppress apoptosis of H9c2 cells following H/R injury. H9c2 cells were divided into control, H/R model, and H/R + acacetin (12.5, 25, and 50 *μ*g/ml) groups. (a–d) Relative protein levels of beclin 1, p62, and LC3-II in each group were measured by Western blot. (e) Immunofluorescence of LC3 II in H9c2 cells as detected by fluorescence microscopy. Bars indicate mean ± standard deviation of three independent experiments. ^#^*P* < 0.05 compared with control group; ^#^*P* < 0.05, ^∗^*P* < 0.01 compared with H/R model group. H/R: hypoxia/reoxygenation; LC3-II: microtubule-associated protein 1A/1B-light chain 3.

Examination of autophagy-linked protein expression by Western blot showed that the accumulation of autophagy markers (including LC3-II, Beclin 1, and p62) elicited by acacetin occurred in a dose-dependent manner (Figure 4(a)). Our experimental results show that acacetin could protect H9c2 cells from H/R injury by promoting autophagy. Compared with the H/R group, acacetin pretreatment for 12 h enhanced the apoptosis-promoting effect of H/R following blocking of autophagy flux, indicating that acacetin induced autophagy as a cytoprotective mechanism.

We further observed that acacetin promoted autophagy, as indicated by the aggregation of Stub-RFP and Sens-GFP on autophagosomes following lentiviral infection (Figure 4(e)). In this experiment, the intensity of red/green spots observed by fluorescence microscopy was used to judge the degree of autophagy of H9c2 cardiomyocytes in each experimental group after H/R. Compared with the H/R group, H9c2 cells treated with acacetin exhibited higher fluorescence intensity, confirming that acacetin induced autophagy in H9c2 cells. Compared with the H/R group, use of acacetin improved the autophagy activity of H9c2 cells.

### 3.5. Acacetin Enhanced Autophagy via the PI3K/Akt Signaling Pathway

**Figure 5:**
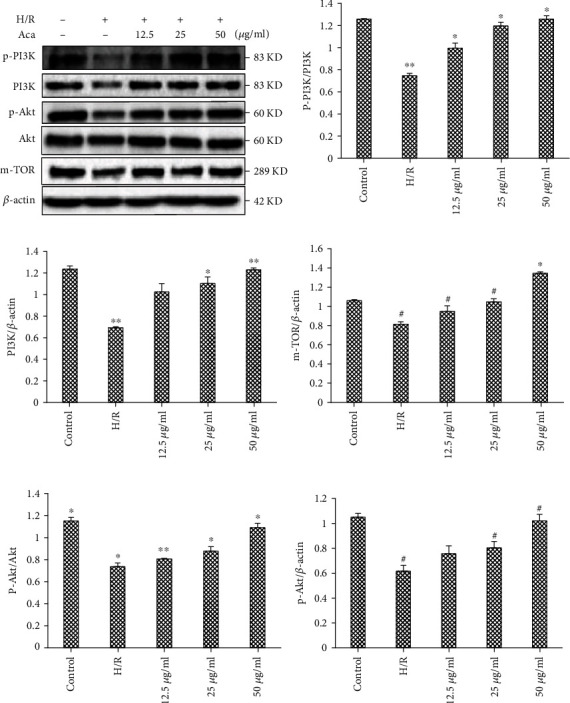
Acacetin mitigates H/R injury by regulating the PI3K/Akt/mTOR signaling pathway. H9c2 cells were divided into control, H/R, H/R + acacetin (12.5, 25, and 50 *μ*g/ml), and H/R +50 *μ*g/ml acacetin + LY294002 groups. (a–f) Relative protein levels of p-Akt, p-PI3K, and mTOR in each group were measured by Western blot. Bars indicate mean ± standard deviation of three independent experiments. ^#^*P* < 0.05 compared with control group; ^#^*P* < 0.05, ^∗^*P* < 0.01 compared with H/R model group. H/R; hypoxia/reoxygenation; p-: phosphorylated; PI3K: phosphoinositide 3 kinase; mTOR: mechanistic target of rapamycin.

The PI3K/Akt/mTOR signaling pathway is the most classical signaling pathway involved in the multiple prolongation mechanism of mTOR. We further verified the effect of acacetin on the expression of PI3K/Akt/mTOR signaling pathway-related proteins in H9c2 cells injured by H/R. Western blot results showed that compared with the control group, expression of PI3K, phosphorylated PI3K (p-PI3K), Akt, and phosphorylated Akt (p-Akt) proteins in the H/R model group were decreased. Indeed, compared with the H/R model group, PI3K, p-PI3K, Akt, and p-Akt gradually increased with increasing concentrations of acacetin (Figure 5). As shown in Figure 5, acacetin activated the PI3K/Akt/mTOR signaling pathway and participated in the protection of cardiomyocytes, thus inhibiting cell metabolism and promoting proliferation.

**Figure 6:**
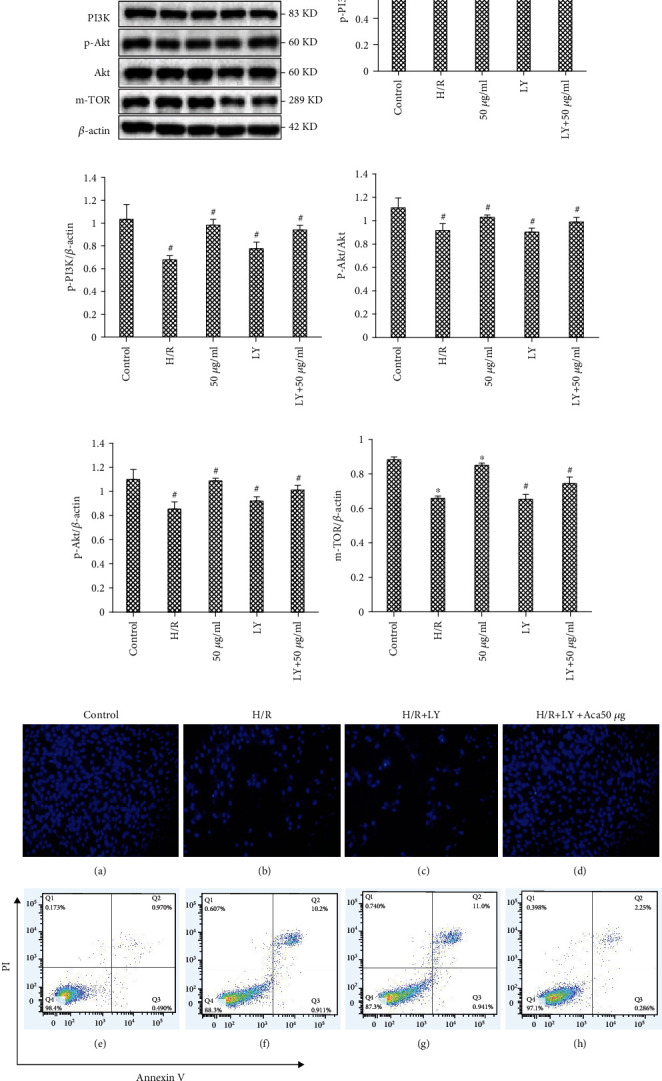
Acacetin induced autophagy in H9c2 cells by inhibiting the PI3K/Akt/mTOR pathway. H9c2 cells were divided into control, H/R model, H/R +50 *μ*g/ml acacetin, H/R + LY294002, and H/R +50 *μ*g/ml acacetin + LY294002 groups. (a–f) Relative protein levels of p-Akt, p-PI3K, and mTOR in each group as measured by Western blot. Bars indicate mean ± standard deviation of three independent experiments. ^#^*P* < 0.05 compared with control group; ^#^*P* < 0.05, ^∗^*P* < 0.01 compared with H/R model group. (g) Cell apoptosis rates of each group were analyzed by flow cytometry. (f) Cell survival rates of each group were analyzed by DAPI staining. DAPI: 4′,6-diamidino-2-phenylindole; H/R: hypoxia/reoxygenation; PI3K: phosphoinositide 3 kinase; mTOR: mechanistic target of rapamycin.

To verify the mechanism of acacetin, we determined whether the PI3K/Akt inhibitor LY294002 (LY, 20 mM) could abolish its activity. Cell viability of every group, as assessed by flow cytometry and DAPI assay, demonstrated that inhibition of PI3K/Akt abrogated the protection elicited by acacetin pretreatment (Figure 6). These results indicate that the efficacy of acacetin was abolished by LY294002.

## 4. Discussion

The objective of the current study was to determine whether the beneficial effect of acacetin on cardiomyocytes exposed to H/R injury involves autophagy as a protective mechanism. In this study, we showed that acacetin preconditioning suppressed myocardial cell apoptosis of rat cardiomyocytes following damage caused by H/R injury. Moreover, acacetin enhanced autophagy by regulating activity of the PI3K/Akt signaling pathway and enhancing the interaction of autophagosomes to maintain a higher level of autophagy, which offered a protective effect against H/R injury.

Acacetin is a natural product with antiproliferative and antioxidant activities. Acacetin has also been shown to exert an antiproliferative effect by blocking cell cycle progression and inducing apoptosis. Moreover, acacetin has been shown to target mitochondria to induce apoptosis of chronic lymphocytic leukemia (CLL) B lymphocytes through increased reactive oxygen species formation, mitochondrial membrane potential collapse, mitochondrial permeability transition, release of cytochrome c, caspase 3 activation, and finally apoptosis, whereas normal healthy B lymphocytes were unaffected. In addition, oral administration of acacetin elicited potent *in vivo* anticancer activity in CLL xenograft mouse models [28]. Our present study found that acacetin preconditioning yielded beneficial effects and protected myocardial cells through activation of autophagy.

Under activating physiological conditions, autophagy can remove useless, superfluous, or damaged cells and organelles to maintain homeostasis of the body [29]. Previous studies revealed that moderate upregulation of autophagy could protect cardiomyocytes against H/R injury, whereas excessive autophagy could aggravate myocardial H/R injury [20, 30, 31]. LC3, p62, and Beclin 1 are central autophagy-related proteins involved in autophagy flux [12]. Our results clearly show that acacetin activated these autophagy-related proteins and increased cell viability, thereby maintaining the homeostasis of cells. During the early stage of autophagy, LC3-I was transformed into membrane LC3-II, leading Stub-RFP and Sens-GFP (red/green colocalized dots) to accumulate on autophagosomes. Our results showed that red/green fluorescence was the weakest in the control group. Compared with the H/R group, fluorescence intensity of the acacetin group was enhanced, indicating that acacetin could effectively activate autophagy in myocardial cells.

As an essential prosurvival pathway, PI3K/Akt signaling plays classical signal transduction roles in the development of cardioprotection against myocardial H/R injury. To further verify that acacetin induced autophagy in H9c2 cells by inhibiting the PI3K/Akt/mTOR pathway, we evaluated the contents of PI3K/Akt/mTOR proteins, which were significantly increased after adding inhibitors (Figure 6) [32, 33]. DAPI and flow cytometry results also indicated increase in cardiomyocyte apoptosis, suggesting that PI3K/AKT/mTOR pathway activation may promote the induction of autophagy in H9c2 cells. In addition, it was concluded that acacetin can inhibit expression of the proapoptotic factor Bax and enhance the expression of antiapoptotic factors Bcl-2 and mTOR, thus reducing apoptosis of H9c2 cardiomyocytes during H/R.

In summary, the results of this study suggest that acacetin inhibited apoptosis through the PI3K/Akt/mTOR signaling pathway to alleviate H/R injury in H9c2 cardiomyocytes by promoting autophagy. Thus, the clinical efficacy and safety of acacetin for reducing myocardial H/R injury warrant further study.

## Data Availability

The data used to support the findings of this study are available from the corresponding author upon request.
